# Elevated Liver Enzymes Can Predict Complications Early After Pancreatic Resection

**DOI:** 10.3390/jcm15082851

**Published:** 2026-04-09

**Authors:** Theresa Hofmann, Imad Kamaleddine, Clemens Schafmayer, Guido Alsfasser

**Affiliations:** Department of General, Visceral, Thoracic, Vascular and Transplantation Surgery, University Hospital Rostock, University of Rostock, Schillingallee 35, 18055 Rostock, Germany; theresa.hofmann@uni-rostock.de (T.H.); imad.kamaleddine@med.uni-rostock.de (I.K.); clemens.schafmayer@med.uni-rostock.de (C.S.)

**Keywords:** pancreatic surgery, complication, elevated liver enzymes, failure to rescue

## Abstract

**Background/Objectives**: Pancreatic surgery has always been associated with a variety of complications. In the current study, we analyzed more than 800 consecutive pancreatic resections and tried to find clinically relevant routine parameters that could predict adverse outcomes at an early stage. We focused on hepato-pancreato-biliary routine parameters, especially on liver enzymes, because so far there are no studies showing any correlation between postoperatively elevated liver enzymes and postoperative complications. **Methods**: All pancreatic resections of a tertiary care center from 2003 until 2025 were documented prospectively and analyzed retrospectively. Data analysis comprised descriptive as well as inferential statistical analyses. **Results**: Laboratory values from 808 consecutive resections were analyzed for the first week after surgery. Elevated aspartate aminotransferase (AST) was associated with postoperative hemorrhage on POD 1, pulmonary insufficiency on POD 1 to 4, other complications on POD 1 to 5, MODS on POD 2 to 4, and development of pneumonia on POD 3 to 5. Elevated alanine aminotransferase (ALT) was associated with pulmonary insufficiency on POD 1 to 4 and POD 6, pneumonia, and other complications on POD 3. It was also associated with MODS on POD 1 to 6. Bilirubin elevated preoperatively and on POD 1 could not really predict any complication. In this study, we can also confirm that elevated amylase and lipase can predict complications. **Conclusions**: This is the first study that shows a correlation between postoperatively elevated AST and ALT and the development of postpancreatectomy complications. Elevated AST and ALT, especially in combination with postoperative pancreatitis or at least elevated pancreatic enzymes, can identify patients at risk for life-threatening conditions and might be useful to decrease failure-to-rescue patients.

## 1. Introduction

Pancreatic surgery has always been associated with a variety of complications [1,2,3]. About 50% of patients suffer from some sort of complication, of which many are easy to handle [4]. Every deviation from the regular clinical course could be considered a complication. For example, the need for additional antibiotic treatment usually leads to a prolonged hospital stay, but most of these patients never experience life-threatening conditions. However, other complications pose a serious clinical challenge. These severe complications very often require revision surgery [5]. In recent years, some specific complications, like delayed gastric emptying, postpancreatectomy acute pancreatitis, postpancreatectomy hemorrhage, and pancreatic fistula, have been clearly defined and graded [6,7,8,9]. Especially, the development of pancreatic fistula has been the focus of many studies, and predictive factors have been analyzed and described [10]. Elevated pancreatic enzymes in the postoperative period were identified as predictors of the development of many complications [11,12,13,14]. For example, the combination of postoperative pancreatic fistula and intra-abdominal abscess formation is a predictor of delayed postpancreatectomy hemorrhage [15]. There are numerous studies evaluating predictors of complications after pancreatic surgery. Addeo et al. described radiologic characteristics on preoperative CT scans that are able to identify so-called acinar contents, and patients were divided into groups with low, moderate, or high acinar content. Patients with high radiological acinar scores have a statistically significantly higher rate of pancreatic-specific complications [16]. Another group analyzed the proportion of different cell types on the cut edge of the pancreas by histology and found that an increased frequency of acinar cells is associated with an increase in immediate postoperative complications [17]. A further group replicated these results and showed that the risk is dichotomous, either low or high, depending on acinar score [18].

In liver surgery, elevated bilirubin poses a serious risk of complicating the postoperative course [19]. A recent retrospective analysis in a single-center study showed that preoperatively elevated bilirubin can increase complications in pancreatic surgery, whereas biliary drainage prevents this effect [20]. However, a recent meta-analysis concluded that routine biliary drainage cannot be recommended [21], and it even showed worse prognosis in a large registry study [22].

In the current study, we analyzed more than 800 consecutive pancreatic resections and tried to find clinically relevant routine parameters that help predict adverse outcomes at an early stage. We focused on hepato-pancreato-biliary routine parameters since these laboratory variables are commonly used in the diagnosis and treatment of pancreatic pathologies. Furthermore, clinical relevance was of the utmost importance. Normal laboratory values cannot—in our opinion—be used as predictors for complications as described by others [23]. Additionally, helpful laboratory values are those measured routinely—for example, within the first days after surgery. We therefore focused on all laboratory values within the first week after pancreatic resection.

## 2. Materials and Methods

All consecutive pancreatic resections performed in a tertiary care center between 2003 and 2025 were documented prospectively and analyzed retrospectively. Documentation was based upon a standardized protocol that was once established and used thereafter for every pancreatic resection. All types of resections and every diagnosis were included in this analysis. Patients gave written informed consent and were interviewed pre- and postoperatively, and during the initial hospital stay, all data were recorded. The study was reviewed and approved by the local ethics committee (approval number: A 2025-0171). We collected and analyzed pre- and postoperative liver function tests, pancreatic enzymes, and biliary parameters, and compared the occurrence of complications. Data were extracted from the database and converted to CSV format. Raw data were then imported into the programming language R (version 4.4.3). Complications and laboratory parameters were identified based on clinical relevance. Data were checked for completeness, and a plausibility check was performed. Then, cut-off values were defined (normal institutional value, ≤2-fold increase, ≤3-fold increase, and ≥3-fold increase), which were used to define groups for further analysis.

Data analysis comprised descriptive as well as inferential statistical analyses.

### 2.1. Descriptive Statistics

Each variable was defined and analyzed descriptively. Metric variables were recorded with mean, standard deviation, median, quartiles, and coefficient of variation. Categorical variables, especially dichotomic variables, were analyzed for absolute and relative frequency.

### 2.2. Inferential Statistics

At first, all categories, including those with three or fewer case numbers, were excluded. Variables with extremely unevenly distributed characteristics (i.e., more than 95% of characteristics in one category) were excluded from interferential analysis and included in descriptive analysis. In the interferential analysis, every postoperative complication was defined as a different group. Further analysis was carried out within these groups based on the above-mentioned cut-off values. *p*-values were calculated for each group and parameter.

Testing of hypotheses was performed using exploratory, bivariate-oriented inferential analysis. Associations between categorical variables and dichotomic variables were analyzed by chi-square test or Fisher’s exact test, where applicable. Metric variables were tested using the *t*-test and the Mann–Whitney U test, where applicable. Multiple testing was corrected using the Bonferroni–Holm correction method.

### 2.3. Defined Complications

Pancreatic fistula and delayed gastric emptying (DGE) were defined according to the International Study Group for Pancreatic Surgery (ISGPS) standard as published previously [6,7]. According to the ISGPS, delayed gastric emptying is defined by the number of days for which a nasogastric tube is required and no solid food can be digested [7]. Postoperative pancreatic fistulas (POPF) are among the most complex but also most common complications in pancreatic surgery, which arise in up to 45% of pancreatic surgeries [6]. While drain amylase levels > 3 times higher than normal amylase serum levels (formerly grade A POPF) are now considered a biochemical leak, classification as clinically relevant POPF requires a relevant change in clinical management (grade B, e.g., need for endoscopic or percutaneous drains, angiographic interventions for bleeding, signs of infection without organ failure, prolonged drainage > 3 weeks). Organ failure and the necessity of revision surgery are defining for grade C POPF [6]. As implicated in this classification, a biochemical leak itself is common and does not necessarily change the clinical course for the patient. Postoperative hemorrhage was defined as any clinical sign of bleeding that required either transfusion, intervention, or operation. Presence of organ failure was extracted from the intensive care unit (ICU) record, and each complication during ICU stay was defined as follows: pulmonary insufficiency was defined as the need for non-invasive ventilation and hypoxia without manifest pneumonia. Pneumonia was defined as pulmonary insufficiency together with radiologic signs of pneumonia/infiltration and microbiologic criteria, with the need for antibiotic therapy. Renal insufficiency was defined as an increase in creatinine of more than 0.3 mg/dL within 48 h or an increase of 1.5-fold from the starting value within 7 days, combined with low urine output, with the need for hemofiltration or dialysis. Sepsis was defined as life-threatening organ dysfunction based on an increase of more than two points in the SOFA score. MODS was the presence of more than one documented organ failure at a given time. All other complications were summarized in one group for statistical purposes. Other complications included are acute urinary retention, lymphatic fistula, anastomotic insufficiency, portal vein thrombosis, vascular problems, infectious complications (abdominal abscess, wound infection, peritonitis), seroma of wounds, perforation, and ileus.

## 3. Results

### Patient Characteristics

We analyzed 808 consecutive pancreatic resections. The patient population consisted of 501 male and 307 female patients. Age at time of surgery ranged from 14 to 88 years. Among all resections, the indication was carcinoma in 436 cases, with 326 pancreatic, 64 periampullary, and 46 distal bile duct carcinoma. A total of 197 patients were resected for chronic pancreatitis. Other indications were, among others, IPMN, any infiltrating carcinoma of the upper GI-tract, metastases, neuroendocrine tumors, benign lesions of the papilla of Vater, and traumatic injuries. The main ASA scores of all patients were 358 times ASA II and 394 times ASA III. The predominant operative procedure was pancreatic head resection in 504 patients with 347 pylorus-preserving pancreatoduodenectomies, 84 classic Whipple procedures, and 73 duodenum-preserving pancreatic head resections. Additionally, 148 distal pancreatectomies, 68 total pancreatectomies, and 88 other resections (enucleation, atypical resection, segmental resection) were performed. For further details, see Table 1.

Based on our statistical analysis, there are several parameters associated with certain complications. We could also see statistically significant results of moderately elevated parameters; however, we only focused on pathologic elevation, which we defined as more than three times higher than the upper institutional limit. In the following section, we try to summarize the statistical association of pathologically elevated routine parameters and complications. Preoperatively elevated amylase was associated with renal insufficiency, and AST with delayed gastric emptying. Preoperative pathologically elevated γGT was associated with the development of pancreatic fistula. Routine values on postoperative days (POD) one to seven that showed statistically significant associations with complications were serum amylase, serum lipase, aspartate aminotransferase, alanine aminotransferase, and, to a lesser extent, serum bilirubin and γGT. Elevated amylase on POD 1 to 3 was associated with the development of pancreatic fistula, on POD 1 and 2 with postoperative hemorrhage, and, if elevated on POD 3, additionally pneumonia, sepsis, and multi-organ dysfunction (MODS). Elevated lipase on POD 1 and 2 was associated with the development of pancreatic fistula and with sepsis. Elevated aspartate aminotransferase (AST) was associated with postoperative hemorrhage on POD 1, pulmonary insufficiency on POD 1 to 4, other complications on POD 1 to 5, MODS on POD 2 to 4, and development of pneumonia on POD 3 to 5. Elevated alanine aminotransferase (ALT) was associated with pulmonary insufficiency on POD 3 and 4, pneumonia, and other complications on POD 3. It was also associated with MODS on POD 1 to 6. Elevated bilirubin was associated with delayed gastric emptying on POD 2 and 5 and the development of shock on days 3 and 4. Preoperatively elevated bilirubin could not predict any complication. However, later in the postoperative course, there was an association between elevated bilirubin on POD 7 and pneumonia, renal insufficiency, and sepsis. Elevated amylase on POD 7 was associated with pneumonia and shock. Postoperatively elevated γGT was not associated with any complication except pulmonary insufficiency on POD 1. For an overview, see Table 2 and Table 3.

## 4. Discussion

Table 1 clearly shows that there are multiple parameters within the first postoperative days that are associated with multiple complications. All parameters are routine parameters, like liver enzymes or pancreatic enzymes, that are commonly used in the assessment of any postpancreatectomy period. Elevated pancreatic enzymes are known to be predictors of complications and postoperative pancreatic fistula [24,25,26,27]. Elevated amylase is associated with increased postoperative burden of complications like Clavien–Dindo ≥ II, with increased rates of bacteraemia, pleural effusion, postpancreatectomy hemorrhage, postpancreatectomy acute pancreatitis, and organ-site infections. The combined occurrence of postoperatively elevated amylase together with pancreatic fistula led to a shorter median time to morbidity [26]. However, so far, no data indicate that postoperatively elevated liver enzymes help predict complications after pancreatic surgery. Preoperative AST and ALT, and especially the De Ritis ratio of AST/ALT, were studied extensively and helped predict various complications. For example, an elevated AST/ALT ratio has been found to be an independent risk factor for mediastinal and pericardial drainage volume in aortic arch surgery [28]. Another systematic review and meta-analysis demonstrated that the preoperative De Ritis ratio predicts a poor prognosis for patients with urothelial carcinoma [29]. Xu et al. analyzed preoperatively elevated De Ritis ratio and its prognostic value in patients after surgery for hepatocellular carcinoma. They found an increased rate of postoperative kidney failure with preoperatively increased De Ritis ratio [30]. The incidence of postoperative kidney failure was analyzed by Gultekin et al. after cardiac surgery. In this study, the authors found that preoperatively as well as postoperatively, an elevated De Ritis ratio was associated with an increased rate of acute postoperative kidney injury [31]. Liu et al. evaluated the preoperative De Ritis score in non-cardiac surgery. They showed that an elevated De Ritis score increased the risk for perioperative ischemic stroke in patients with type 2 diabetes [32]. Of course, the prognostic value of these enzymes was evaluated in liver surgery. Abdelhadi et al. showed that elevated AST and ALT on postoperative day three were valuable prognostic indicators for posthepatectomy liver failure [33]. Vassanasiri et al. described the need for an early warning parameter for posthepatectomy liver failure, because by definition, laboratory values on or after postoperative day five are needed for diagnosis. The authors could show that an elevation of AST and ALT on day one after surgery was predictive of liver failure. However, cut-off values were more than three times higher than the upper limit. Moreover, this study clearly shows the ability of liver enzymes to be an early predictor after surgery [34]. There are many reports on liver enzymes in the context of pancreatic surgery, but none to our knowledge has convincingly shown the ability to predict certain complications as published for pancreatic enzymes. Xiao et al. conducted a retrospective study of histopathologically confirmed pancreatic ductal adenocarcinoma and used ALT to correlate survival after pancreatic resection. However, the authors did not evaluate the possible negative effect of ALT elevation [35]. Huang et al. evaluated CA19-9 as a predictor for survival after pancreatic resection for malignancy. The authors claimed that obstructive jaundice could interfere with the predictive value of CA19-9; therefore, they used several liver enzymes for further analysis. They could show a better prediction of CA19-9 in patients with obstructive jaundice, when ratios were calculated between this tumor marker and certain liver enzymes [36]. Kapoor et al. analyzed the effect of preoperatively deranged AST and ALT on immediate postoperative outcome. They could show that preoperative elevation of AST in multivariate analysis was independently predictive of 30-day postoperative mortality. However, no information on postoperative elevation and especially on correlation with complications is described [37]. Winter et al. characterized routinely ordered perioperative laboratory tests after pancreaticoduodenectomy. Using multivariate logistic regression, they identified biochemical markers for morbidity and mortality. The most significant markers were further evaluated in detail. Among all significant multivariate predictors, aminotransferase was identified as a predictor for postoperative death. However, significant predictors for postoperative complications were blood urea nitrogen, albumin, and postoperative amylase. No liver enzymes were identified as a predictor of postoperative complications [38]. Hughes et al. evaluated the correlation of preoperative liver function tests and postoperative complications. Interestingly, only 37% of all 452 pancreaticoduodenectomies had documented complications. In univariate analysis, they demonstrated that low or normal preoperative aspartate aminotransferase and alkaline phosphatase had higher rates of complications. In multivariate analysis, they could confirm this effect with alkaline phosphatase [23]. In our study, we intentionally disregarded any statistical effect of low or normal laboratory values, because from our point of view, possible mechanisms of predicting complications cannot be established. Hackert et al. could show that liver ischemia—recognized by elevated liver enzymes—could lead to a fatal prognosis after pancreatic head resection [39]. Futugawa et al. investigated the effects of chronic hepatic dysfunction, which included liver cirrhosis, on the outcome after pancreatic resection. This multi-institutional retrospective study identified elevated serum aspartate aminotransferase as a significant risk factor for hepatic decompensation. This, in turn, had a negative influence on the overall outcome. However, no correlation with pancreas-specific complications was shown [40].

From a clinical standpoint, we hesitate to draw much attention to γ-glutamyl transferase and alkaline phosphatase because these parameters are often elevated after manipulation of the bile ducts, for example, after performing a bilio-digestive anastomosis. So, early postoperative increases often occur naturally and are not distinguishable from complications. However, an elevation three times the upper normal limit was defined as pathologic elevation and was therefore included in our analysis; it did show correlation with the development of pancreatic fistula on postoperative days 2 and 3. Some parameters in our analysis were statistically significant on single days only, like elevated bilirubin on days 2 and 5, predicting delayed gastric emptying. These findings are hard to explain, and the clinical implications of this finding are difficult to establish. On the other hand, it might be a useful tool to identify patients at risk. However, early identification of delayed gastric emptying does not necessarily rescue patients from a life-threatening condition but could identify patients at risk for possible pulmonary complications due to reflux or aspiration. Indeed, elevated bilirubin on postoperative day 7 is associated with pneumonia, but there is no correlation with pulmonary insufficiency at any other time point. Other parameters in our analysis did show increased rates of complications when elevated below three times the upper normal limit. A recent comprehensive review showed that mildly elevated liver enzymes can occur in up to 30% of patients after laparoscopic cholecystectomy. However, the authors did not find any adverse clinical outcomes [41]. Tan et al. showed that postoperative transient elevation of liver enzymes could occur in laparoscopic cholecystectomy but not in open cholecystectomy [42]. In our study, we could not assess any threshold in the subgroups of patients with moderately elevated parameters due to the small sample size. However, any correlation with complications is very hard to interpret, because in these groups every increase “up to threefold” could include marginally elevated values as well as almost threefold increased values. Therefore, from a clinical perspective, we only looked at the parameters that were increased more than three times the upper normal limit to clearly identify pathologically elevated parameters. Normal or slightly elevated preoperative parameters [23] are weak predictors from a clinical standpoint, and any correlation is also hard to explain.

Elevation of aspartate aminotransferase and alanine aminotransferase is an indicator of liver damage at a cellular level [43,44]. Obviously, liver damage increases the susceptibility to acquiring complications, as described above. Elevated postoperative amylase and lipase, and therefore the development of postoperative acute pancreatitis, is certainly a challenging clinical problem after pancreatic surgery [8]. However, if pathologically elevated postoperative pancreatic enzymes and liver enzymes are combined, we might be able to identify the patients that do not only acquire a postoperative pancreatic fistula but to a higher probability also suffer from pulmonary and renal failure. Therefore, those patients with elevated pancreatic enzymes and concurrently elevated liver enzymes could be identified as being at high risk for developing life-threatening complications after pancreatic resection. Even postpancreatectomy hemorrhage is associated with elevated aspartate aminotransferase on postoperative day 1 or elevated bilirubin on postoperative day 3 in our study. Elevated liver enzymes can also predict multiorgan failure, as shown with elevated alanine aminotransferase on postoperative days 1–6 and elevated aspartate aminotransferase on postoperative days 2–4. Additionally, elevated aspartate aminotransferase on postoperative days 1 to 5 was associated with the occurrence of any other complication, which worsens clinical outcomes as well. These complications could be infections and any other complications described above. Unfortunately, we cannot establish correlations to specific complications due to small sample sizes. Another clinically challenging problem, of course, is the failure to rescue some patients after pancreatic resection [45,46], because life-threatening complications could not be treated early enough. However, the routine parameters we used can at least help to identify patients at risk. This could, in turn, lead to an earlier start of intensive care treatment or closer monitoring and potentially prevent severe complications or even death. As mentioned earlier, there are significant parameters like elevated bilirubin on POD 7 as a predictor of pneumonia. However, elevated bilirubin has no other association with pulmonary insufficiency. Therefore, this finding is also hard to explain, but was discussed above. Sometimes significant values need to be discussed for plausibility, or random effects must be ruled out, which could occur as a consequence of certain statistical methods like multiple testing [47,48]. Therefore, we added a Bonferroni–Holm correction. For all these parameters, more studies are needed to confirm their predictive value. However, in our current study, we analyzed routine parameters that were prospectively collected in a large cohort of more than 800 pancreatic resections. We not only analyzed these parameters using standard statistical procedures but also interpreted them from a clinical standpoint, and all possible effects of non-elevated values were ignored. Overall, we could show that increased liver enzymes within the first postoperative week after any pancreatic resection could pose a significant risk for developing severe complications. Together with routine parameters that have been identified in many previous studies, our findings will contribute to developing further strategies for reducing failures to rescue patients after pancreatic surgery.

## 5. Summary and Conclusions

Thus, elevated liver enzymes and cellular liver damage indicate a negative outcome of the postoperative course after pancreatic resection. Elevated liver enzymes after pancreatic resection can help predict complications. Elevated AST and ALT, especially in combination with postoperative pancreatitis or at least elevated pancreatic enzymes, can enable us to identify patients at risk of life-threatening conditions and will be useful to decrease failure-to-rescue rates.

## Figures and Tables

**Table 1:** Patient characteristics.

entire sample	808 patients (100%)
age distribution	14–88 years
gender distribution	female: 307 (38.00%) male: 501 (62.00%)
preoperative risk assessment/ ASA–score (American Society of Anesthesiologists)	ASA 1 15 (1.86%) ASA 2 358 (44.30%) ASA 3 394 (48.80%) ASA 4 20 (2.48%)
indication	carcinomas total: 436 (53.96%) pancreatic malignancy: 326 (40.35%) papillary/peri-ampullary carcinoma; adenoma: 64 (7.92%) bile duct carcinomas: 46 (5.69%) chronic pancreatitis: 197 (24.38%) others: 175 (21.68%) IPMN, metastases, insulinoma, gastrinoma, leiomyosarcoma, benign lesions of the ampulla of Vater, liposarcoma, malignant duodenal carcinoid, primary duodenal carcinoma, B-cell Lymphoma, choledochal-jejunal fistula, ruptured lienalis aneurysma with pancreatic ischemia, infiltrating stomach cancer or kidney cancer, trauma
procedures	pancreatic head resections total: 504 (62.38%) classic Whipple operation: 84 (10.40%) pylorus-preserving pancreatic head resection: 347 (42.95%) duodenum-preserving pancreatic head resection: 73 (9.04%) distal pancreatectomy: 148 (18.32%) pancreatectomy: 68 (8.42%) others: 88 (10.89%) (atypical resections, segmental resections, enucleations)
histology	benign: 334 (41.3%) malignant: 456 (56.4%) borderline tumor: 19 (2.3%)

**Table 2:** Overview of timepoints when parameters predict complications for up to one week after surgery. Results from inferential analysis. All parameters were elevated more than three times the upper normal limit. For statistical details, see Table 3.

	Pre-Op	POD 1	POD 2	POD 3	POD 4	POD 5	POD 6	POD 7
Postoperative Pancreatic Fistula, POPF		Amylase	Amylase	Amylase				
		Lipase	Lipase					
	γGT		γGT					
Delayed Gastric Emptying, DGE			Bilirubin			Bilirubin		
	AST							
Postpacretectomy Hemorrhage, PPH		Amylase	Amylase					
		AST						
				Bilirubin				Bilirubin
Pneumonia				Amylase				Amylase
		Lipase						
				AST	AST	AST		
				ALT				
								Bilirubin
Pulmonary Insufficiency		AST	AST	AST	AST			
				ALT	ALT			
		γGT						
Renal Insufficiency	Amylase							
	AST	AST	AST					
			ALT	ALT				
								Bilirubin
Multiple Organ Dysfunction Syndrome, MODS				Amylase				
			AST	AST	AST			
		ALT	ALT	ALT	ALT	ALT	ALT	
Sepsis				Amylase				
		Lipase		Lipase				
								Bilirubin
Shock								Amylase
				Bilirubin	Bilirubin			
Other	Amylase							
		AST	AST	AST	AST	AST		
				ALT				

**Table 3:** Detailed statistical analysis of significant parameters after Bonferroni–Holm correction.

Variable	Parameter/Timepoint	*p*-Value	Absolute Risk (%)	Relative Risk	Odds Ratio
Postpancreatectomy Hemorrhage	Amylase POD 1	0.03	0.36	1.4	1.62
	Amylase POD 2	0.045	0.41	1.36	1.6
	AST POD 1	0.011	0.35	1.52	1.79
	Bilirubin POD 3	0.032	0.46	1.81	2.5
	Bilirubin POD 7	0.032	0.64	2.17	4.28
Delayed Gastric Emptying	AST preop	0.003	0.14	2.05	2.22
	Bilirubin POD 2	0.024	0.18	2.28	2.55
	Bilirubin POD 5	0.018	0.31	5.6	7.65
Postoperative Pancreatic Fistula	Amylase POD 1	0.003	0.45	4.12	6.7
	Amylase POD 2	0.003	0.55	2.73	4.82
	Amylase POD 3	0.003	0.64	2.73	5.86
	Lipase POD 1	0.004	0.57	2.92	5.5
	Lipase POD 2	0.006	0.65	2.59	5.55
	µGT preop	0.021	0.24	0.7	0.6
Other	Amylase preop	0.011	0.18	0.6	0.51
	AST POD 1	0.009	0.45	1.44	1.8
	AST POD 2	0.005	0.49	1.6	2.18
	AST POD 3	0.005	0.56	1.65	2.5
	AST POD 4	0.009	0.68	1.75	3.33
	AST POD 5	0.025	0.73	1.96	4.53
	ALT POD 3	0.043	0.47	1.49	1.92
Multiple Organ Dysfunction Syndrome	Amylase POD 3	0.048	0.2	2.09	2.37
	AST POD 2	0.003	0.17	4.13	4.75
	AST POD 3	0.003	0.26	5.29	6.78
	AST POD 4	0.003	0.39	5.82	8.94
	ALT POD 1	0.008	0.13	2.34	2.55
	ALT POD 2	0.006	0.17	3.38	3.89
	ALT POD 3	0.006	0.21	5.75	6.99
	ALT POD 4	0.008	0.23	4.3	5.3
	ALT POD 5	0.008	0.28	5.02	6.59
	ALT POD 6	0.042	0.3	3.17	4.08
Pneumonia	Amylase POD 3	0.012	0.22	2.46	2.87
	Amylase POD 7	0.028	0.43	5.54	8.94
	Lipase POD 1	0.011	0.16	2.39	2.66
	AST POD 3	0.006	0.21	3.03	3.57
	AST POD 4	0.036	0.29	2.5	3.11
	AST POD 5	0.039	0.36	3.2	4.55
	ALT POD 3	0.015	0.17	2.63	2.96
	Bilirubin POD 7	0.047	0.29	3.43	4.4
Pulmonary Insufficiency	AST POD 1	0.029	0.01	1.99	2.17
	AST POD 2	0.004	0.25	3.33	4.1
	AST POD 3	0.004	0.34	3.97	5.49
	AST POD 4	0.004	0.46	3.55	5.67
	ALT POD 3	0.032	0.23	3.23	3.92
	ALT POD 4	0.005	0.35	2.87	3.86
	µGT POD 1	0.049	0.06	0.61	0.59
Renal Insufficiency	Amylase preop	0.039	0.01	0.38	0.37
	AST POD 1	0.003	0.11	2.97	3.21
	AST POD 2	0.003	0.15	4.28	4.85
	AST POD 3	0.003	0.18	3.97	4.61
	ALT POD 2	0.034	0.13	3.33	3.67
	ALT POD 3	0.032	0.14	4.45	5.02
	AP POD 4	0.041	0.3	3.95	5.21
	Bilirubin POD 7	0.015	0.29	5.71	7.6
Shock	Amylase POD 7	0.006	0.86	3.35	17.44
	Bilirubin POD 3	0.004	0.51	2.74	4.58
	Bilirubin POD 4	0.021	0.52	2.54	38.73
Sepsis	Amylase POD 3	0.021	0.22	2.33	2.71
	Lipase POD 1	0.002	0.22	3.37	4.04
	Lipase POD 3	0.002	0.8	7.93	35.64
	AP preop	0.009	0.01	0.14	0.13
	Bilirubin POD 7	0.022	0.36	3.73	5.24

## Data Availability

The original contributions presented in this study are included in the article. Further inquiries can be directed to the corresponding author.

## Abbreviations

The following abbreviations are used in this manuscript:

AST	Aspartate Aminotransferase
ALT	Alanine Aminotransferase
POD	Postoperative Day
POPF	Post Operative Pancreatic Fistula
ISGPS	International Study Group for Pancreatic Surgery
ICU	Intensive Care Unit
MODS	Multi-Organ Dysfunction Syndrome
DGE	Delayed Gastric Emptying
